# 

**Published:** 1883-03

**Authors:** 


					﻿INTERNATIONAL HAHNEMANNIAN ASSOCIATION.
Bureau of Obstetrics and Diseases of Women and Children.
W. H. Leonard, M. D., Minneapolis, Some of the Causes of Can-
cel* of the Uterus.	,
John Hall, M. D., Toronto, Membranous Dysmenorrhoea.
Edward Mahoney, M. D., Liverpool,
George F. Foot, M. D., Stamford, Ct., Diseases of Children.
Edward G. Rushmore, M. D., Plainfield, N. J., Clinical Observa-
tions.
Adolph Fellger, M. D., Philadelphia,
L. A. Rendell, M. D., New Haven, Ct;,
J. R. Haynes, M. D., Chairman, Indianapolis, Retroverted and
Adhered Uterus and Treatment.
NOTICE.
All articles for publication, books for review, business communications, etc.,
must be sent to Editor, 2109 Chestnut Street, Philadelphia.
CONTRIBUTORS:
Drs. H. C. Allen, T. F. Allen, Edward Bayard, J. B.Bell,' E. W/ Berridge,
Geo. H. Clark, A- C; Cowperthwaite. Wm. J. Guernsey, W. A, Hawley, 1
John Hall, W. M. James, W. H. Jenny, Ad. Lippe, C. Lippe,
Malcolm MacFarlan,C. F. Nichols, C. Pearson, T. F. Pom-
eroy, Leila A. BenDell, C. Carleton Smith, p. P. Wells, -
. ‘ Wni. P. Wesselhoeft, David Wilson >(London). ’
T. P. Wilson, and many, others.
■ Authors of accepted papers are furuished with copies of the
number in which their articles appear-; application for such copies
to be made when sending papers. Any irregularity’in the receipt
of this Journal should be promptly reported to Editor.'
				

